# Pleasure as a Vaso-Dilator

**Published:** 1902-10-25

**Authors:** 


### Hospital Clinics and Medical Progress.

### PLEASURE AS A VASO-DILATOR.

As was to be expected from Dr. Ferrier, who last Saturday delivered the Harveian Oration before the Royal College of Physicians, his discourse dealt with the nervous system; while, again, as surely was appropriate to the occasion, it also dealt with the heart and circulation. Dr. Ferrier chose for his subject some of the principal relations of the heart to the nervous system which have been learnt since the time of Harvey. After giving a short account of the innervation of the heart and blood vessels, and after showing how any alteration of blood pressure influenced the circulation in the cerebrum, Dr. Ferrier proceeded to discuss the complementary problem how far disturbance of cerebral action, and more especially how far states of feeling influenced the heart and blood-vessels. The most manifest organic expressions of emotion were, he said, those which were to be observed in the domain of the circulation. Every feeling, whether agreeable or painful, acted primarily as an excitant and tended to disturb the existing balance of the vaso-motor apparatus. Whether the quality of the sensation or emotion as such was always associated with characteristic and uniform changes in the circulation was still a disputed point, but the balance of the evidence favoured the view that pleasurable emotions and sensations were accompanied by vascular dilatation and low tension, the contrary being associated with vascular constriction and high tension pulse. Thus we were brought to a point of very great importance, for it would seem that pleasurable and painful emotions are not merely subjective sensations of consciousness, but are at the same time objective conditions of exalted or depressed vital energy respectively, showing themselves not only in outward attitude and gesture but in the relative power of the organism to withstand debilitating agencies of all kinds. Nor was this all, for Dr. Ferrier showed that there was a similarity if not identity between the effects produced on the circulation by painful and pleasurable emotions and by painful and pleasurable sensations. Thus we come round to many suggestive ideas in regard to therapeutics. We have long endeavoured by varying the arterial tension to modify cerebral activity, can we then by modifying cerebral activities in the direction of pleasure rather than of pain, so act upon cardio-vascular tension as to modify nutrition, not in the brain alone but throughout the organism? Here we see new light upon the therapeutic effects of change, travel, and pleasure-seeking, new explanations of the benefits of a pleasurable massage, new therapeutic advantages in a jolly dinner and a funny play. It is demoralising, no doubt, but clearly there is more to be said than some may think in favour of the pursuit of pleasurable emotions by those whose arteries are getting stiff.

